# Loss of vimentin expression in preoperative biopsies independently predicts poor prognosis, lymph node metastasis and recurrence in endometrial cancer

**DOI:** 10.1038/s44276-024-00105-2

**Published:** 2024-10-18

**Authors:** Marta E. Hjelmeland, Hilde E. Lien, Hege F. Berg, Kathrine Woie, Henrica M. J. Werner, Frédéric Amant, Ingfrid S. Haldorsen, Jone Trovik, Camilla Krakstad

**Affiliations:** 1 Centre for Cancer Biomarkers, Department of Clinical Science, University of Bergen, Bergen, Norway; 2 Department of Gynecology and Obstetrics, Haukeland University Hospital, Bergen, Norway; 3 Department of Obstetrics and Gynecology, Maastricht University Medical Center (MUMC+), Maastricht, The Netherlands; 4 GROW-Research Institute for Oncology and Reproduction, Maastricht, The Netherlands; 5 Division Gynecologic Oncology, UZ Leuven, Leuven, Belgium; 6 Department of Clinical Medicine, University of Bergen, Bergen, Norway; 7 Mohn Medical Imaging and Visualization Centre, Department of Radiology, Haukeland University Hospital, Bergen, Norway

## Abstract

**Background:**

Precise preoperative risk classification of endometrial cancer is crucial for treatment decisions. Existing clinical markers often fail to accurately predict lymph node metastasis and recurrence risk. Loss of vimentin expression has emerged as a potential marker for predicting recurrence in low-risk endometrial cancer patients. We assessed whether vimentin expression in preoperative biopsies predicts poor prognosis and lymph node metastasis in a large multicentre cohort.

**Methods:**

Vimentin expression was evaluated using immunohistochemistry in 1483 patients diagnosed with endometrial cancer across 14 hospitals in Europe. Expression levels of vimentin were analyzed in conjunction with clinical characteristics for predicting disease-specific survival and lymph node metastases.

**Results:**

Vimentin loss was significantly associated with aggressive disease and poor survival. Adjusted for clinicopathological variables, vimentin remained independently prognostic with a hazard ratio (HR) of 1.68 (95% CI 1.16–2.42, *P* = 0.006). Vimentin expression remained independently prognostic in endometrioid endometrial cancer- and FIGO staged 1 patient. Interestingly, vimentin loss independently predicted lymph node metastases, with an HR of 1.83 (95% CI 1.13–2.95, *P* = 0.014).

**Conclusions:**

Loss of vimentin in preoperative biopsies serves as an independent predictor of poor prognosis and lymph node metastases. Incorporating vimentin as a clinical marker can improve risk stratification and treatment decisions.

## Introduction

Endometrial cancer, the most common gynecological malignancy in high-developmental index countries, exhibits a favorable prognosis with a 5-year overall survival rate of 86% [1]. However, endometrial cancer is one of the few malignancies showing a rapid increase in both incidence and mortality rates [2]. Diagnosis and treatment include histological examination of preoperative tissue, often complemented by imaging to stratify patients into low-, intermediate- and high-risk groups [3–5]. Primary treatment is surgery, with adjuvant chemotherapy offered to the high-risk group [6]. Surgical staging is performed according to the International Federation of Gynecology and Obstetrics (FIGO) and serves as a strong prognostic marker in endometrial cancer [7]. In 2023, the staging system was updated to incorporate lymphovascular space invasion and the four molecular subgroups; POLE ultramutated, Mismatch-repair deficient (MMRd)/microsatellite instable (MSI), copy-number high (or p53 abnormal) and copy-number low (no specific molecular profile) [5]. However, the global implementation of molecular characterization is challenging. Specifically, obtaining data on POLE mutations, which requires Sanger sequencing, is costly and technically demanding when working with limited preoperative tissue samples. Additionally, the final FIGO stage is determined based on surgicopathological findings, first being determined postoperatively, and thus not used to guide surgical decisions and early patient stratification [8]. To address these challenges, incorporating immunohistochemically based independent risk markers becomes crucial. By identifying new biomarkers, clinicians can more effectively stratify patients early in the treatment process, aiding decision-making, and patient outcome.

Patients with lymph node metastases face an overall worse prognosis, highlighting the importance of early identification of these individuals. Surgical removal of lymph nodes, lymphadenectomy, is routinely performed on high-risk patients. This procedure carries an increased risk of operative complications, which can significantly impact the quality of life [9, 10]. Recently, regional lymphadenectomy is more often replaced with the less invasive sentinel lymph node (SLN) evaluation [11]. However, SLN evaluation requires highly skilled surgeons, and expensive instruments and poses additional challenges in severely obese patients. Alternative methods supporting risk stratification from preoperative samples can help identify patients at high risk of recurrence and detect lymph node metastases and poor prognosis [12].

The main preoperative predictive factors utilized to assess tumor characteristics include histologic type, grade, depth of myometrial invasion, and tumor size. However, identifying and integrating additional preoperative molecular biomarkers could enhance characterization, especially when SLN assessment is unavailable. There has been a strong focus on discovering preoperative prognostic markers in endometrial cancer. Notably, estrogen receptor (ER), progesterone receptor (PR), and tumor suppressor p53 have demonstrated strong prognostic value and are currently being integrated into clinical practice [4, 13]. Additional prognostic markers have been suggested, including L1CAM and HER2 [14–16]. However, these markers are not universally applicable across all subgroups of endometrial cancer. In fact, approximately 10–15% of patients initially classified with low-intermediate disease experience recurrence whilst 50% of high-risk patients do not [17–20]. We are therefore still in need of additional markers to be able to cover all subsets of endometrial cancer patients.

Vimentin, a type III intermediate filament expressed in mesenchymal cells, is widely known for its role in the epithelial-to-mesenchymal transition (EMT). Elevated vimentin expression has been associated with metastatic potential and poor prognosis in various cancer types, including liver, breast, lung, and prostate [21–29]. Interestingly, we recently identified loss of vimentin as a potential marker for recurrence in endometrial cancer, including for tumors confined to the uterus (FIGO stage 1) [30]. Our findings contradict the previously reported function of vimentin in cancer, suggesting that vimentin may have a tissue-specific function in the endometrium.

The aim of this study was to validate that loss of vimentin expression serves as a robust marker of recurrence in endometrial cancer. Additionally, we investigate whether assessing vimentin expression in preoperative samples could help improve the identification of patients with high-risk disease, including lymph node metastases, in a large international multicentre patient series [13].

## Materials and methods

### Patient series

Preoperative biopsies from 1483 primary endometrial cancers were collected in a multicenter study from 14 centers between 2001 and 2019 [13]. Samples were prepared as formalin-fixed and paraffin-embedded (FFPE) specimens at the respective centers. Clinicopathological information including age at primary treatment, FIGO 2009 stage, histological type and grade of primary tumor, and follow-up data were retrieved from medical records as previously described [1]. All included patients gave written and informed consent. The study has been approved by the Western Regional Committee for Medical and Health Research Ethics (REK 2015/594) according to Norwegian legislation and regulations. Samples are stored in Bergen Biobank for gynecological cancer (REK 2014/1907) and clinicopathological data and follow-up information are stored in the Bergen Gynecological Cancer Health Registry (approved by the Norwegian Data Inspectorate 2016/7421 and Regional Ethical Committee, REK 7226).

### Immunohistochemistry (IHC)

FFPE tissue was used to generate tissue microarray (TMA) from collected samples as previously described [31]. Briefly, the tumor area with the highest tumor cell content was identified on hematoxylin and eosin-stained slides. Three tissue cylinders (0.6 mm) were punched out of the donor block and mounted in a recipient paraffin block, using a custom-made precision instrument (Beecher Instruments, Silver Spring, MD, USA). TMA slides were deparaffinized in xylene and re-hydrated in graded ethanol before microwave boiling in antigen retrieval solution (pH 9, S2367, Dako, Glostrup, Denmark) for 15 min, followed by peroxidase blocking (S2023, Dako, Glostrup, Denmark) for 8 min at room temperature. The TMA slides were incubated with vimentin (D21H3, Cell Signaling Technology, MA, USA) diluted 1:300 for 60 min at room temperature, followed by 30 min incubation with secondary anti-rabbit antibody (K4003, Agilent Technologies, Santa Clara, USA). Slides were developed for 6 min with diaminobenzidine peroxidase (DAB) (K3468, Envision detection system, Dako, Glostrup, Denmark) and counterstained using hematoxylin (S3301, Dako, Glostru, Denmark) before dehydration and mounting.

### Evaluation of staining

Only epithelial cells were considered when evaluating vimentin staining. For each patient, a semi-quantitative staining index (SI) system was used to calculate the product of staining intensity (0 = negative, 1 = weak, 2 = moderate, 3 = strong) and area of positive cells (0 = 0%, 1 = less than 10%, 2 = 10–50%, 3 = more than 50% of tissue with positive staining). Evaluation of staining was performed blinded for clinical characteristics and outcome and expression was evaluated as an average of three cylinders. Evaluation was not assessed in cylinders where the tumor component was low. For the evaluation of disease-specific survival, cases were ranked by staining index. Cases with SI: 1–9 were combined based on similarities in survival and defined as “vimentin positive”. Cases with SI: 0, corresponding to loss of vimentin was defined as “vimentin loss”. A subset of 384 samples was evaluated by two independent observers (MEH, HEL) blinded for patient characteristics and outcomes to investigate inter-observer reproducibility. The kappa value for vimentin in the two groups was 0.76.

### Molecular classification

Molecular subgroup classification was defined using the Proactive Molecular Risk Classifier for Endometrial Cancer (ProMisE) [32]. Briefly, *POLE-*ultramutated tumors were identified by detection of mutations in exons 9, 11, 13, or 14 of *POLE*, the catalytic subunit of DNA polymerase-ε using Sanger sequencing. In tumors with no detected POLE mutation, IHC was used to determine MMR deficiency by loss of expression in one or more of the MMR proteins, MLH1, MSH2, MSH6, and PMS1 [33]. The copy-number low group was defined as *POLE* wild type, MMR proficient, and TP53 wild type whilst tumors with abnormal p53 expression (loss or overexpression) by IHC were defined as copy-number high.

### Statistical analysis

Statistical analyses were performed using IBM SPSS Statistics for Macintosh (Version 29.0.2.0, IBM Corp, Armonk, NY, USA). All statistical tests were two-sided with a level of significance set at *P*-values < 0.05. Agreement between the two methods was evaluated using Cohen´s Kappa statistics. Associations between two categorical groups were evaluated using the Chi-squared test. Survival analysis for disease-specific survival was performed using the Kaplan–Meier method, and differences between the two groups were compared using the Log-rank (Mantel–Cox) test. Disease-specific survival was defined as the time of primary surgery to death due to endometrial cancer. Patients who died of other causes or lost during follow-up time were censored. Cox´s proportional hazard regression modeling was used for multivariate survival analyses and lymph node metastases. Due to the high correlation between estrogen receptor (ER) and progesterone receptor (PR) expression, these variables were merged into one covariate, ER/PR status. ER and PR status in patients from 2001 until 2015 were defined as previously described [13]. Patients after 2015 were defined as low with ER and/or PR expression <30%. The interaction term was included in the Cox regression model if the interaction was observed.

## Results

### Loss of vimentin expression in preoperative biopsies is associated with aggressive disease and poor outcome in endometrial cancer

**Fig. 1:**
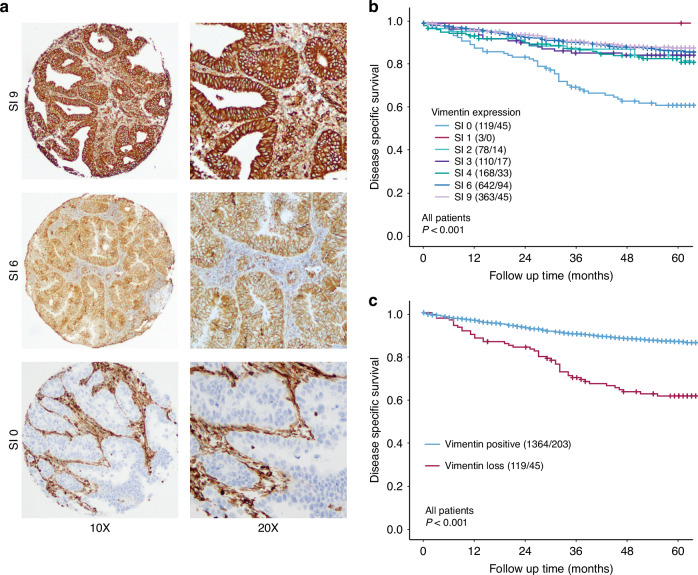
Loss of vimentin expression is associated with poor survival in endometrial cancer. Representative brightfield images (10× and 20×) of staining index (SI) score, SI 9, 6, and 0 by immunohistochemical staining of vimentin protein expression (**a**). Disease-specific survival for individual SI-scores (**b**). Loss of vimentin is significantly associated with poor survival in all endometrial cancer patients (**c**). Kaplan–Meier survival curves presented with a number of cases/number of disease-specific deaths. *P*-values from Mantel–Cox Log-rank test.

Vimentin expression in preoperative samples from 1483 women diagnosed with endometrial cancer was evaluated by immunohistochemistry using a staining index (SI), ranging from strong expression (SI 9) to loss of expression (SI 0) (Fig. 1a). Vimentin expression was observed in 92% (*n* = 1364) of the samples while complete loss of expression was seen in the remaining 8% (*n* = 164). Raw data was analyzed for association with disease-specific survival (DSS) (Fig. 1b). Patients with SI 1–9 had similar prognosis with a 5-year DSS ranging from 1.0 to 0.88. Patients with SI 0 had a worse prognosis with a 5-year DSS of 0.62. For all further analyses, SI values were grouped in loss (SI = 0) and positive (SI 1–9) expressions. Patients with loss of vimentin expression had significantly reduced disease-specific survival compared to patients with positive vimentin expression (5-year DSS 0.62 vs 0.85, Log-rank *P* < 0.001, Fig. 1c).

Loss of vimentin in preoperative samples was significantly associated with clinicopathological markers of aggressive disease, including high age at diagnosis, high FIGO stage, and non-endometrioid histologic subtype (Chi-square, *P* < 0.001, Table 1). Markedly, patients with loss of vimentin were more often diagnosed with deep myometrial infiltration (≥50%, Chi-share, *P* < 0.001), lymph node metastases (Chi-square, *P* = 0.001), and disease recurrence (Chi-square, *P* < 0.001).

**Table 1:** Vimentin protein expression in curettage samples is associated with clinicopathological variables in endometrial cancer patients.

Variable	Vim pos *n* (%)	Vim loss *n* (%)	*P*-value
Number of patients	1364	119	
Age			**<0.001**
<66	666 (95)	37 (5)	
≥66	698 (89)	82 (11)	
FIGO-09 stage^a^			**<0.001**
I–II	1147 (93)	83 (7)	
III–IV	212 (86)	35 (14)	
Histologic type^a^			**<0.001**
Endometrioid	1134 (95)	66 (5)	
Serous	122 (82)	26 (18)	
Clear cell	41 (73)	15 (27)	
Carcinosarcoma	46 (85)	8 (15)	
Undifferentiated	19 (83)	4 (17)	
Histologic grade^a,b^			0.529
Grade 1	535 (95)	26 (5)	
Grade 2	422 (93)	32 (7)	
Grade 3	165 (95)	8 (5)	
Myometrial infiltration^a^			**<0.001**
<50%	806 (95)	43 (5)	
≥50%	452 (88)	60 (12)	
Recurrence^a,c^			**<0.001**
No	1063 (94)	67 (6)	
Yes	214 (86)	35 (14)	
Lymph node metastasis^a^			**0.001**
No	818 (93)	64 (7)	
Yes	120 (84)	22 (16)	

*FIGO* International Federation of Gynaecologist and Obstetrics, *n* number of patients, *Vim pos* positive vimentin expression, *Vim loss* loss of vimentin expression.

Positive: SI 1–9, Loss: SI 0.

Statistically significant *P*-values (<0.05) are bold.

^a^Data missing on FIGO stage for 6 patients, histologic type for 1 patient, myometrial infiltration for 122 patients, and lymph node metastatic status for 459 patients.

^b^Endometrioid histology only.

^c^104 patients with metastasis at primary treatment are censored.

### Loss of vimentin identifies aggressive disease and poor prognosis in subgroups of endometrial cancer

**Fig. 2:**
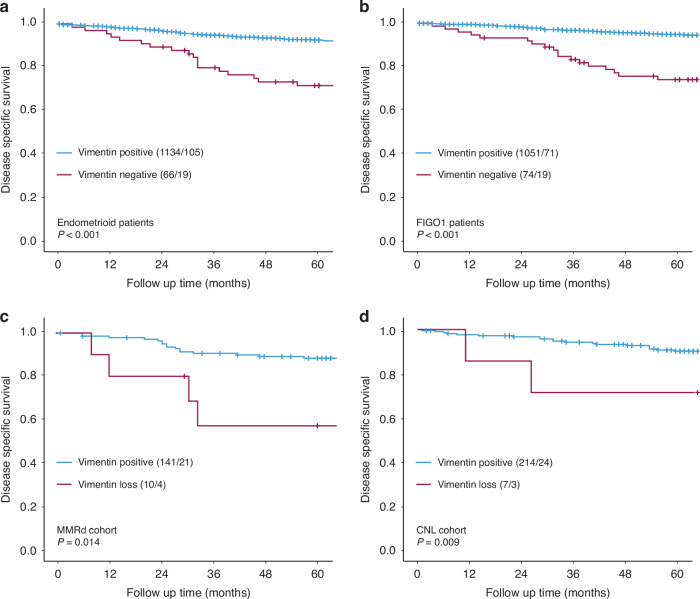
Disease-specific survival in defined subgroups of endometrial cancer according to vimentin expression. Loss of vimentin expression in preoperative samples is significantly associated with worse outcomes in the defined subgroups of patients with endometrioid endometrial histology (**a**), tumors confined to the uterus (FIGO stage 1) (**b**), mismatch-repair deficient (MMRd) tumors (**c**), and copy-number low (CNL) tumors (**d**). Numbers in brackets: number of patients in the group/number of events in the group. *P*-values from Mantel–Cox Log-rank test.

In the subgroup of tumors with endometrioid histology, 94% (*n* = 1134) expressed vimentin, while the remaining 6% (*n* = 66) showed a loss of vimentin expression. Loss of expression was significantly associated with clinicopathological variables of aggressive disease, including high age, deep myometrial infiltration (≥50%), recurrence, and lymph node metastases (Chi-square, *P* < 0.05, Table 2). Notably, patients with endometrioid histology who had loss of vimentin expression also experienced a significantly worse prognosis compared to those with positive expression (5-year DSS: 0.71 vs 0.91, Log-rank, *P* < 0.001, Fig. 2a).

**Table 2:** Loss of vimentin in preoperative specimens in endometrioid and FIGO stage 1 tumors associated with clinicopathological features of aggressive disease.

	Endometrioid patients			FIGO 1 patients		
Variable	Vim pos *n* (%)	Vim loss *n* (%)	*P*-value	Vim pos *n* (%)	Vim loss *n* (%)	*P*-value
Number of patients	1134	66		1051	74	
Age			**0.011**			**0.023**
<66	595 (96)	24 (4)		541 (95)	28 (5)	
≥66	539 (93)	42 (7)		510 (92)	46 (8)	
FIGO-09 stage^a^			0.063			
I–II	1009 (95)	53 (5)				
III–IV	124 (91)	12 (9)				
Histologic grade^a^			0.529			**<0.001**
Grade 1	535 (95)	26 (5)		493 (95)	24 (5)	
Grade 2	422 (93)	32 (7)		337 (94)	22 (6)	
Grade 3	165 (95)	8 (5)		213 (88)	28 (12)	
Histologic type						**<0.001**
Endometrioid				937 (95)	49 (5)	
Serous				61 (82)	13 (18)	
Clear cell				16 (70)	7 (30)	
Carcinosarcoma				27 (84)	5 (16)	
Undifferentiated				10 (100)	0 (0)	
Myometrial infiltration^a^			**<0.001**			**<0.001**
<50%	695 (97)	22 (3)		740 (96)	35 (4)	
≥50%	375 (91)	37 (9)		298 (89)	37 (11)	
Recurrence^a,b^			**<0.001**			**<0.001**
No	943 (96)	43 (4)		917 (95)	52 (5)	
Yes	148 (89)	19 (11)		124 (85)	21 (15)	
Lymph node metastasis^a^			**0.024**			
No	700 (95)	37 (5)				
Yes	72 (89)	9 (11)				

*FIGO* International Federation of Gynaecologist and Obstetrics, *n* number of patients, *Vim pos* positive vimentin expression, *Vim loss* loss of vimentin expression.

Positive: SI 1–9, Loss: SI 0.

Statistically significant *P*-values (<0.05) are bold.

Endometrioid patients: ^a^Data missing on FIGO stage for 2 patients, histologic grade for 12 patients, myometrial infiltration for 71 patients, and lymph node metastatic status for 382 patients. ^b^47 patients with metastasis at primary treatment is censored.

FIGO 1 patients: ^a^Data missing on histologic grade for 8 patients and myometrial infiltration for 15 patients. ^b^11 patients with metastasis at primary treatment is censored.

Similarly, within the subgroup of patients with no detectable extra-uterine tumor spread (FIGO stage 1), 93% (*n* = 1051) of the samples expressed vimentin, while the remaining 7% (*n* = 74) had loss of expression. In FIGO stage 1 patients, loss of vimentin was again significantly correlated with parameters of aggressive disease, including high age, high grade, non-endometrioid histology, deep myometrial infiltration (≥50%), and recurrence (Chi-square, *P* < 0.05, Table 2). Furthermore, loss of vimentin was a strong predictor of poor survival (5-year DSS 0.74 vs 0.93, Log-rank, *P* < 0.001, Fig. 2b).

As the field of defining endometrial cancer molecular subgroups evolves for prognostication and treatment stratification, we explored whether loss of vimentin could identify patients with poor prognosis within the defined molecular subgroups. Among 502 patients with full molecular classification, 5% (*n* = 27) exhibited loss of vimentin expression (Supplementary Table 1). Vimentin expression did not correlate with any specific molecular subgroup. However, loss of vimentin was significantly associated with worse disease-specific survival both in the MMRd subgroup (5-year DSS: 0.60 vs 0.85, Log-rank, *P* = 0.01, Fig. 2c) and the CNL subgroup (5-year DDS: 0.57 vs 0.88, Log-rank, *P* = 0.009, Fig. 2d). Loss of vimentin was also significantly associated with poor prognosis within the subgroup of endometrioid CNL patients (*n* = 200; Log-rank, *P* = 0.026).

### Loss of vimentin expression in preoperative samples independently predicts poor survival, lymph node metastasis, and recurrence

To investigate the added value of vimentin in a preoperative setting, multivariate analyses were performed. Loss of vimentin expression showed independent prognostic impact in Cox survival analysis, adjusted for age, curettage histology, and ER/PR status, with a hazard ratio (HR) of 1.68 (95% CI 1.16–2.42, *P* = 0.006, Table 3). Additionally, vimentin expression independently predicted poor prognosis within patients with endometrioid histology, with HR 2.57 (95% CI 1.46–4.51, *P* = 0.001, Table 4). Interestingly, loss of vimentin expression in patients with FIGO stage 1 tumors was a stronger predictor of prognosis with an HR of 2.34 (95% CI 1.29–4.27, *P* = 0.005, Table 5) compared to ER/PR status with an HR of 1.47 (95% CI 0.89–2.45, *P* = 0.135). Furthermore, vimentin expression independently predicted lymph node metastases after adjusting for age and curettage histology in a multivariate analysis with an HR of 1.83 (95% CI 1.13–2.95, *P* = 0.014, Table 6).

**Table 3:** Prediction of poor disease-specific survival based on age, curettage histology, ER/PR, and vimentin protein expression in preoperative samples in endometrial cancer patients.

Risk factor	*N*	Univariate HR (95% CI)	*P*-value	Multivariate HR (95% CI)	*P*-value
Age (mean = 66 years)	1239	1.06 (1.05–1.07)	**<0.001**	1.04 (1.03–1.06)	**<0.001**
Curettage histology^a^					
Low risk	931	1	**<0.001**	1	**<0.001**
High risk	308	5.50 (4.18–7.24)		5.05 (3.25–7.83)	
ER/PR status^b^					
Normal	817	1	**<0.001**	1	**<0.001**
Low	422	3.34 (2.54–4.40)		2.44 (1.60–3.72)	
Vimentin expression					
Positive	1146	1	**<0.001**	1	**0.006**
Loss	93	2.98 (2.08–4.27)		1.68 (1.16–2.42)	

Statistically significant *P*-values (<0.05) are bold.

^a^Curettage histological classification: low risk (endometrioid grade 1 and 2) or high risk (endometrioid grade 3 or non-endometroid).

^b^Low ER/PR expression: loss of or low expression of ER and loss of PR expression.

Events: 211.

**Table 4:** Univariate and multivariate analysis in endometrioid endometrial cancer patients.

Risk factor	N*	Univariate HR (95% CI)	*P*-value	Multivariate HR (95% CI)	*P*-value
Age (mean = 66 years)	1007	1.05 (1.03-1.07)	**<0.001**	1.05 (1.03–1.07)	**<0.001**
Curettage histology^a^					
Low risk	887	1	**<0.001**	1	**<0.001**
High risk	120	4.29 (2.85–6.44)		5.16 (3.00–8.89)	
ER/PR status^b^					
Normal	768	1	**<0.001**	1	**0.009**
Low	239	2.06 (1.40–3.04)		1.93 (0.88–2.10)	
Vimentin expression					
Positive	955	1	**<0.001**	1	**0.001**
Loss	52	3.22 (1.87–5.56)		2.57 (1.46–4.51)	

^*^Endometrioid cases only, events: 110.

Statistically significant *P*-values (<0.05) are bold.

^a^Curettage histological classification: low risk (endometrioid grade 1 and 2) or high risk (endometrioid grade 3 or non-endometroid).

^b^Low ER/PR expression: loss of or low expression of ER and loss of PR expression.

**Table 5:** Prediction of poor disease-specific survival based on age, curettage histology, ER/PR, and vimentin protein expression in FIGO stage 1 endometrial cancer patients.

Risk factor	N*	Univariate HR (95% CI)	*P*-value	Multivariate HR (95% CI)	*P*-value
Age (mean = 66 years)	945	1.07 (1.04–1.09)	**<0.001**	1.05 (1.03–1.08)	**<0.001**
Curettage histology^a^					
Low risk	778	1	**<0.001**	1	**<0.001**
High risk	167	4.11 (2.61–6.47)		2.68 (1.60–4.47)	
ER/PR status^b^					
Normal	686	1	**<0.001**	1	0.135
Low	259	2.47 (1.58–3.86)		1.47 (0.89–2.45)	
Vimentin expression					
Positive	887	1	**<0.001**	1	**0.005**
Loss	58	3.76 (2.10–6.70)		2.34 (1.29–4.27)	

^*^FIGO stage 1 cases only, events: 78

Statistically significant *P*-values (<0.05) are bold.

^a^Curettage histological classification: low risk (endometrioid grade 1 and 2) or high risk (endometrioid grade 3 or non-endometroid).

^b^Low ER/PR expression: loss of or low expression of ER and loss of PR expression.

**Table 6:** Prediction of lymph node metastasis based on age, curettage histology, and vimentin protein expression in endometrial cancer patients.

Risk factor	N*	Univariate HR (95% CI)	*P*-value	Multivariate HR (95% CI)	*P*-value
Age (mean = 66 years)	981	1.03 (1.02–1.05)	**<0.001**	1.02 (1.00–1.04)	**0.016**
Curettage histology^a^					
Low risk	718	1	**<0.001**	1	**<0.001**
High risk	263	3.56 (2.52–5.01)		3.10 (2.18–4.42)	
Vimentin expression					
Positive	901	1	**<0.001**	1	**0.014**
Loss	80	2.63 (1.65–4.20)		1.83 (1.13–2.95)	

^*^Endometrial cancer patients with available data for all variables included in the univariate analysis, events: 134

Statistically significant *P*-values (<0.05) are bold.

^a^Curettage histological classification: low risk (endometrioid grade 1 and 2) or high risk (endometrioid grade 3 or non-endometroid).

## Discussion

This is to our knowledge the largest study of vimentin expression in endometrial cancer. We report in a large multicentre study, that 8% of women diagnosed with endometrial cancer exhibit loss of vimentin expression in preoperative samples. We found a significant association between loss of vimentin expression and features of aggressive disease and unfavorable prognosis in both univariate and multivariate analyses. Notably, vimentin remains a robust predictor of survival within specific subgroups, such as within endometrioid endometrial tumors and FIGO stage 1 disease. Our analyses reveal that loss of vimentin expression in preoperative samples independently predicts lymph node metastasis in multivariate models. These findings indicate that vimentin may serve as a valuable preoperative marker in endometrial cancer, especially to detect low-risk tumors that may later recur.

Our finding is in stark contrast to the general role of vimentin as a key marker of epithelial-to-mesenchymal transitions (EMT). Previous studies investigating the impact of vimentin expression find that overexpression is associated with metastatic disease and poor prognosis in liver, breast, lung, and prostate cancers [21, 22, 24–28]. Vimentin is an intermediate type III filament normally expressed in mesenchymal cells. Extensive research has revealed that vimentin overexpression in epithelial cells leads to decreased cell-adhesion properties and a mesenchymal phenotype. This transition occurs through the action of transcriptional factors such as Slug and ZEB2, ultimately promoting tumor invasion and metastases via EMT [29, 34, 35]. An additional robust feature of vimentin is the formation of a cage-like network that surrounds the nucleus and other organelles, providing mechanical stability within the cell [36, 37]. Studies have revealed that vimentin acts as a protective shield during cell migration through narrow pores, safeguarding the nucleus from deformation and DNA damage. Interestingly, cells lacking vimentin have been reported to migrate faster through confined spaces [38]. Furthermore, experiments using mouse embryonic fibroblasts demonstrate that loss of vimentin significantly enhances cell motility, particularly in 3D environments and curved capillaries [39]. The stiffness and deformability of cancer cells play a critical role in their capacity to migrate. Additionally, the ability of cancer cells to proliferate is crucial for establishing metastatic lesions at distant sites. EMT-inducing factors have been associated with decreased proliferative capacity [40, 41]. Notably, loss of vimentin has been demonstrated to significantly induce proliferation in several cancer cell lines and promote tumor growth in xenograft in vivo models through increased phosphorylation of AKT and upregulation of β-catenin signaling [42]. This may suggest that loss of vimentin regulates cell spread by controlling nucleus shape and volume, while also contributing to a more proliferative and aggressive phenotype. However, the functional role of vimentin in endometrial cancer remains unknown, necessitating further functional studies.

A favorable prognosis of retained vimentin expression in endometrial cancer has also been reported previously by us and others [30, 43, 44], supporting our findings. However, the patient cohorts in the external studies are varied, ranging from 50 to 341 patients and vimentin expression was only studied in post-operative tissue. A major strength of our study is the large collected multicenter cohort of 1483 endometrial cancer patients, representing all histological subtypes with detailed clinical information and extended follow-up time.

Our study is the first to report that vimentin is an independent predictor of poor prognosis and lymph node metastasis. Moreover, assessing vimentin expression via immunohistochemistry represents a widely utilized and cost-effective approach, and vimentin expression correlates with other preoperative parameters easily available for surgeons, making it a valuable biomarker. Additionally, vimentin protein expression is already an attractive biomarker as it is routinely used in many pathology labs to distinguish between endometrial and endocervical cancer [45, 46]. Effective endometrial cancer management relies on precise preoperative risk assessment to guide treatment decisions, and it is critical to identify patients at risk of lymph node metastases and recurrence. While selection criteria for lymphadenectomy and SLN procedures vary across institutions, either one procedure is typically recommended for patients with high-risk disease based on preoperative histopathological parameters [6]. Thus, there is a strong need for reliable preoperative markers to identify so far non-identified high-risk patients that should also undergo lymphadenectomy/SLN, particularly for those initially classified with low-risk disease. Vimentin could potentially serve as a preoperative marker to identify patients who should undergo lymph node staging. Loss of vimentin expression in preoperative biopsies significantly associates with lymph node metastases within all endometrial cancer patients, as well as endometrioid tumors. Interestingly, we also report that vimentin could serve as a prognostic marker within the molecular subgroup of MMRd and CNL patients. Currently, high-risk MMRd patients are offered dostarlimab in combination with chemotherapy [47]. However, no additional treatment strategy is available for CNL patients. Our results suggest that vimentin may serve as a marker for stratifying patients within the CNL group, also only within the endometrioid subgroup. Specifically, CNL patients with loss of vimentin expression, regardless of histologic grade and presumed stage should undergo lymphadenectomy/SLN evaluation. However, the number of patients in this group is small and should be validated in a larger cohort.

In conclusion, we here present data from a large collection of preoperative samples from endometrial cancer patients showing that loss of vimentin expression predicts lymph node metastases and poor prognosis. Loss of vimentin is a robust biomarker both when including the full cohort and within the subgroup of endometrioid endometrial histological subtype or tumors confined to the uterus (FIGO 1). Vimentin status in endometrial cancer may add valuable prognostic information to better risk stratify patients.

## Supplementary information

Supplementary information

## Data Availability

Detailed datasets generated and/or analyzed during the current study are provided by the corresponding author on reasonable request, pending approval by the local ethical committee.
