# Supplementary material for: Loss of vimentin expression in preoperative biopsies independently predicts poor prognosis, lymph node metastasis and recurrence in endometrial cancer
Source: BJC Rep. 2024 Oct 18;2:81. doi: 10.1038/s44276-024-00105-2 (PMC11524127; doi:10.1038/s44276-024-00105-2)
Supplement: Supplementary file 1 — Supplementary information [file 44276_2024_105_MOESM1_ESM.docx]

**Supplementary Table 1. Vimentin expression detected at same frequencies in the molecular subgroups of endometrial cancer**

| **Variables** | **Vim pos n (%)** | **Vim loss n (%)** | ***P*-value** |
| --- | --- | --- | --- |
| Number of patients | 475 | 27 |  |
| Molecular group |  |  | 0.226 |
| POLE | 43 (98) | 1 (2) |  |
| MMRd | 141 (93) | 10 (7) |  |
| CNL | 214 (97) | 7 (3) |  |
| CNH | 77 (89) | 9 (11) |  |

*Vim* *pos* positive vimentin expression, *Vim loss* loss of vimentin expression, Positive: SI 1-9, Loss: SI 0.

*POLE* POLE ultramutated, *MMRd* Mismatch repair deficiency, *CNL* copy-number low, *CNH* copy-number high
